# 

**Published:** 1911-11-11

**Authors:** 


### Answers to Correspondents.

### ACCIDENT TO A PARISH NURSE.

"A Correspondent" writes: Our parish nurse was cycling in the afternoon (not going to or coming from a case). Her employers (a Samaritan fund) are insured in respect of her against the liability imposed on them by the Workmen's Compensation Act. The insurance company now refuse to pay a claim for an accident and a fortnight's incapacity. I shall be glad to know if you think it possible to make them pay.

Upon the facts stated it is clear that the nurse's employers are not liable to her under the Compensation Act. It follows that the insurance company cannot be forced to pay. The nurse was cycling on her own account, not in discharge of her duties; and that alone would be justification for saying that the accident did not arise "out of and in the course of her employment." It is not necessary, in order to answer this query, to discuss the difficult question whether if she had been going to or returning from a patient at the time she would have been entitled to compensation.

### BOOKS RECEIVED.

Mills and Boon, Ltd.
"Nerves and the Nervous." By Edwin Ash, M.D.

John Wright and Sons, Ltd.
"Dental Anæsthetics." By W. E. Alderson, M.D.

The Ambrose Co., Ltd.
"Demoniacal Obsession and Possession." By Dr. C. Williams.

### Buildings Contemplated and in Progress.

Dearnley.—A new Nurses' Home is being erected at the Dearnley Workhouse Infirmary from the design of Mr. Herbert Clough, of Rochdale. The cost is about £5,000, and forty persons will be accommodated.

Derbyshire.—The erection of a new and extensive asylum is contemplated in the Hope Valley by the County Council of Derbyshire. Some influential persons have raised an objection to the position, but this has received its death-blow from Mr. C. W. Fryer, the nearest landowner to the site, who has publicly withdrawn his opposition; it is interesting also to note that the farmers are not supporting the opposition.

Hull.—Plans for the erection of a new hospital have been prepared by the architect to the Board of Guardians, Mr. J. B. Atkinson. Accommodation is provided for a total of 189 patients at an estimated cost of about £75 per bed. The plans are to be submitted to the Local Government Board.

Prestwich.—The erection of a new Infirmary at the Asylum is contemplated. Mr. Thos. Chadwick, A.R.I.B.A., of Manchester is the architect. Tenders are now being considered.

### Institutional Housekeeping.

ANSWERS TO CORRESPONDENTS.

### FLORENCE NIGHTINGALE MEMORIAL.

WE are happy to be able to report that this Memorial is progressing daily to a satisfactory completion. In another column we publish a letter from Lord Pembroke which, as the *Daily Mail* well says, "comes with singular gracefulness from the son of that 'perfect knight and gallant gentleman' Sidney Herbert of Lea, who devised, organised, and carried through Miss Nightingale's heroic mission to the Crimean hospitals. There is no finer episode in British history than the story of her labours at Scutari, where her magnetic presence brought comfort and hope to our wounded soldiers, and her efforts in a few months reduced the death rate from 42 per cent. to 2 per cent."

We are indebted to many hospital officers for their kind co-operation in connection with this memorial. Mr. Rea, the secretary of King Edward VII. Hospital, Cardiff; Mr. F. Smith, secretary of the Warneford, Leamington and South Warwickshire General Hospital; Mr. Charles Lupton, treasurer of the Leeds General Infirmary; and Mr. Hammick, chairman of the Salisbury Infirmary are consulting their committees and officers. We are especially indebted to Mr. Howard Collins, house governor of the General Hospital, Birmingham; Mr. J. J. Barron, secretary-superintendent of the Royal Infirmary, Bradford; Dr. Watson Williams, of Bristol; Mr. Eugene E. Street, of the West Sussex, East Hants and Chichester General Infirmary; Mr. Harry Johnson, house governor of the Leicester Infirmary; Mr. Arthur Moore, secretary of the County Hospital, Lincoln; Mr. Allen Naldrett, secretary-superintendent of the Royal Southern Hospital, Liverpool; Mr. H. J. Eason, secretary of the Manchester Children's Hospital; Mr. Roden Orde, house governor and secretary of the Royal Victoria Infirmary, Newcastle; and Sister Mary P. Thomson, managing sister of the Royal Infirmary, Sunderland, for taking the lead in organising a centre of collection for each of the districts in which their hospital is situated.

In addition to the amounts received last week, we have the pleasure to acknowledge the following contributions, which are for a statue unless otherwise indicated:—

| | £ | s. | d. |
|---|---|---|---|
| Frank Debenham, Esq. | 5 | 5 | 0 |
| Miss Adelaide Watt | 5 | 0 | 0 |
| R. J. Bowerman, Esq. | 2 | 2 | 0 |
| J. D. Hedderwick, Esq. | 2 | 2 | 0 |
| Jas. Macfarlane, Esq. | 2 | 2 | 0 |
| Miss M. Shrive | 1 | 1 | 0 |
| "M. E. H." (for annuities for nurses) | 1 | 1 | 0 |
| Miss M. I. Burdett | 1 | 0 | 0 |
| Miss A. Burn-Bailey | 1 | 0 | 0 |
| Received by G. Q. Roberts, Esq., Hon. Sec.: | | | |
| Walter Morrison, Esq. | 100 | 0 | 0 |
| W. H. Foster, Esq. | 25 | 0 | 0 |
| Lord Tredegar | 20 | 0 | 0 |
| Col. Sir R. W. Inglis, V.D. | 10 | 10 | 0 |
| Sir Charles Morrison-Bell, Bart. | 10 | 10 | 0 |
| Miss M. Dow, Montreal | 10 | 0 | 0 |
| Sir Thos. Glen Coats, Bt., M.P. (for annuities for nurses) | 10 | 0 | 0 |
| Geo. Cadbury, Esq., J.P. | 5 | 5 | 0 |
| Vivian Gray & Co. | 5 | 5 | 0 |
| Miss Helen James | 5 | 5 | 0 |
| Robert Lodge | 5 | 5 | 0 |
| Mr. and Mrs. J. Muller | 5 | 5 | 0 |
| Miss F. E. Winstanley | 5 | 5 | 0 |
| Charles Davis, Esq., M.V.O. | 5 | 0 | 0 |
| Miss J. Dow, Montreal | 5 | 0 | 0 |
| Mrs. Goodlake | 5 | 0 | 0 |
| Lady Mather (for annuities for nurses) | 5 | 0 | 0 |
| Miss L. K. Palairet | 5 | 0 | 0 |
| "R. M. A." | 5 | 0 | 0 |

Further contributions may be sent to G. Q. Roberts, Esq., Honorary Secretary, Florence Nightingale Memorial, St. Thomas's Hospital, Albert Embankment, London, S.E., or to The Editor of THE HOSPITAL, 29 Southampton Street, Strand, London, W.C.

### WHERE TO WINTER IN ENGLAND.

Those who want a holiday cure in the sunshine, without the expense of a visit abroad, may be interested in the prospectus of the South Devon Health and Holiday Resort, Huntley, Bishop's Teignton, near Teignmouth. This claims the advantages of a country house with those of a modern hydropathic establishment. Sea and moorland air provide the climate, while Turkish and other baths may be taken in the house. The terms for board, residence, and ordinary attendance are three guineas a week, and all particulars may be obtained from Mr. C. F. Carpenter, the proprietor and superintendent.

### The Week's Novelties.

### SANATOGEN AND FORMAMINT.

The worth of a Grand Prix depends chiefly upon the character of the exhibition which confers it. In the case of the great International Hygienic Exhibition, held at Dresden this year, the honour is a perfectly genuine one, and represents a consensus of scientific opinion. It is interesting to note, therefore, that a Grand Prix has been awarded in the pharmaceutical section at this exhibition to Messrs. A. Wulfing and Company for their preparations, Sanatogen, Formamint, and Albulactin, all of which have won high credentials from the medical profession.

### APOLLINARIS WATER.

(The Apollinaris Co., Ltd., 4 Stratford Place, Oxford Street, W.)

The fact that Apollinaris has received the Grand Prix at the recent Dresden Exhibition is a proof of the continued excellence of this high-quality table-water, under modern methods of bottling, which ensure absolute cleanliness and purity. The water is taken from the well-known spring with which tourists in the Ahr Valley are so familiar, and as the water bears transportation very well, those who use it may rely on getting it on the table in a form indistinguishable from that which is served at the Apollinaris restaurants themselves.

### The Hospital.

*SUBSCRIPTION FORM.*

*Please send me* "THE HOSPITAL" *for* ........ *months,*

*commencing with your issue of*..........................., *for*

*which please find enclosed*............................

*Name*.......................................................

*Address*....................................................

..................................

Date.......................................

*To*......................................*Newsagent,*

..................................

RATES OF SUBSCRIPTION (payable in advance).

UNITED KINGDOM.

| | | |
|---|---|---|
| Three Months (including Postage) | ... ... ... ... ... | 2s. 0d. |
| Six Months | do. ... ... ... ... ... | 4s. 0d. |
| Twelve Months | do. ... ... ... ... ... | 6s. 6d. |

FOREIGN AND COLONIAL.

| | | |
|---|---|---|
| Three Months (including Postage) | ... ... ... ... ... | 2s. 6d. |
| Six Months | do. ... ... ... ... ... | 5s. 0d. |
| Twelve Months | do. ... ... ... ... ... | 8s. 8d. |

This form may be sent to any Newsvendor, Railway Bookstall, or to THE MANAGER, "THE HOSPITAL," 28/9, Southampton Street, Strand, W.C.

